# IFUCISTRATEGY: A Spanish Survey on the Management of Invasive Fungal Infection (IFI) in Critically Ill Patients

**DOI:** 10.3390/jof12050339

**Published:** 2026-05-05

**Authors:** Rafael Zaragoza, Ángel Estella, Xavier Nuvials, Mireya Robles-Plaza, Araceli Casado-Gómez

**Affiliations:** 1 Unidad de Cuidados Intensivos, Hospital Universitario Doctor Peset, 46017 Valencia, Spain; zaragoza_raf@gva.es; 2 Fundación Micellium, 46183 Valencia, Spain; 3 Unidad de Cuidados Intensivos, Hospital Universitario de Jerez, 11408 Cádiz, Spain; 4 Departamento de Medicina y Cirugía, Universidad de Cádiz, 11003 Cádiz, Spain; 5 INiBICA, 11009 Cádiz, Spain; 6 Servei de Medicina Intensiva, Hospital Universitari Vall d’Hebron, 08035 Barcelona, Spain; xavier.nuvials@vallhebron.cat; 7 Sepsis Organ Dysfunction and Resuscitation (SODIR), Vall d’Hebron Institut de Recerca (VHIR), 08035 Barcelona, Spain; 8 Vall d’Hebron Barcelona Hospital Campus, 08035 Barcelona, Spain; 9 Pharmacoeconomcis & Outcomes Research Iberia (PORIB), 28224 Madrid, Spain; mrobles@porib.com

**Keywords:** invasive fungal infection, critically ill patients, antifungal resistance, aspergillosis, candidiasis

## Abstract

Background: The objective was to identify management strategies of IFI in critically ill patients through a Spanish national survey. Methods: A cross-sectional multicentre survey among ICU specialists, experienced in IFI, was performed (22 April–25 July 2024). The survey consisted of 13 questions with four closed answers. Results: Sixty-three specialists from 51 hospitals of 16 regions completed the survey. 95% stated that, in high-risk patients with clinical suspicion of Pulmonary Aspergillosis (PA), galactomannan in BAL is performed to guide treatment. In the treatment of patients with PA and influenza, 86% declared that isavuconazole and liposomal amphotericin B are recommended treatments and in high suspicion of *Aspergillus* coinfection, 76% recommended empirical treatment waiting for microbiological confirmation. 90% declared that the use of Extracorporeal Membrane Oxygenation (ECMO) and Renal Replacement Therapies (RRT) could be associated with lower azole levels. Regarding intra-abdominal candidiasis, 78% that physiopathological changes in critically ill patients, reduce their entry into peritoneal fluid. Conclusions: The majority of the respondents agreed (>80%) on: In suspicion of PA, Galactomannan in BAL to guide treatment is mandatory; In case of aspergillosis and influenza, isavuconazole and liposomal amphotericin B are the recommended treatments; The use of ECMO and RRT could be associated with lower azole levels.

## 1. Introduction

Currently, available estimates of fungal disease incidence and mortality remain uncertain. Since 2013, the Leading International Fungal Education (LIFE) portal has facilitated the estimation of the burden of serious fungal infections (FIs) on a country-by-country basis. According to these estimates, more than 300 million people suffer from systemic FIs every year worldwide. Of these cases, 6.5 million people are affected by an immediately life-threatening fungal disease, and around 2.5 million people die from an FI [1,2]. In addition, some pathogenic fungi benefit from climate change, gradually adapting to higher temperatures and becoming more frequent and possibly more virulent. Thus, an increase in invasive fungal infections (IFIs) is expected in the coming years [3].

Additionally, the global burden of IFIs has shown an upsurge in recent years due to the increasing number of immunocompromised patients suffering from various diseases. The role of early and accurate diagnosis in the aggressive containment of IFIs at the initial stages becomes crucial, thus preventing the development of this life-threatening situation in some cases. Over the past decade, a change has been observed in the clinical profile of patients with invasive aspergillosis in the ICU, even surpassing that of the neutropenic immunocompromised patient. Non-neutropenic patients with virus-induced acute respiratory distress syndrome, treated with corticosteroids, as well as patients receiving immunomodulatory therapies, are identified as being at risk for invasive fungal infection [4]. Timely diagnosis of IFIs is necessary to prevent the high morbidity and mortality associated with systemic FI, as late diagnosis always equates with a poor prognosis [5].

Recent advancements have improved our understanding of invasive fungal diseases, leading to revised definitions and diagnostic criteria [6]. However, the diagnostic challenges in intensive care unit (ICU) patients remain unresolved, highlighting the need for further research and evidence generation [7]. Both invasive candidiasis (IC) and invasive aspergillosis (IA) remain important problems in critical ill patients, due to the emergence of new risk factors and species, the growing use of new extracorporeal technologies and new devices, the increasing resistance, and the real need for new diagnostic techniques capable of serving as the future gold standards [8]. IC is the most prevalent form of invasive fungal disease in non-neutropenic ICU patients, presenting as candidemia and deep-seated candidiasis [9]. On the other hand, IA predominantly manifests as invasive pulmonary aspergillosis (IPA) in ICU patients, often associated with comorbidities and respiratory deterioration in viral pneumonia [10]. Antifungal management involves tailored therapy based on guidelines and individual patient factors. The complexity of diagnosing and managing invasive fungal diseases in ICU patients underscores the importance of ongoing research and the need for updated diagnostic criteria and treatment approaches [7].

In addition to the introduction of new drugs, we hope that the coming years will see the creation of multidisciplinary packages of measures designed to reduce the incidence of FIs, improve diagnostic tools and reduce both morbidity and mortality of these patients [5,8].

In Spain, significant gaps persist in the epidemiological characterization of IFIs and their clinical management. Previous initiatives, such as the IFISTRATEGY project, a Spanish national survey focused on hemato-oncologic patients, have provided valuable insights into diagnostic and therapeutic practices in high-risk patients [11]. However, data regarding critically ill patients in ICU settings remain scarce.

To address this gap, we conducted this survey with the aim of understanding the current situation and the strategies used to diagnose and manage IFIs in critically ill patients, according to Spanish intensive care specialists.

## 2. Materials and Methods

A national, multicenter, cross-sectional survey was conducted across various centers within the Spanish national health system (NHS) with intensive care specialists experienced in infectious diseases in critically ill patients serving as respondents.

Data collection was carried out between 22 April and 25 July 2024 through an online survey developed using the Microsoft Forms platform (https://forms.office.com; accessed on 21 April 2024). The survey questionnaire, designed for intensive care specialists, comprised 13 items, each with four predefined answer options. In most cases, respondents were able to select more than one answer.

Subsequently, a descriptive analysis of the responses was conducted. Accordingly, frequency distributions and their corresponding percentages were calculated based on the total number of respondents.

Multiple-choice questions that allow more than one answer are presented using bar charts. The number of respondents who selected each option was calculated and divided by the total number of respondents in the survey. Therefore, the sum of the percentages does not equal 100%.

## 3. Results

A total of sixty-three ICU specialists from fifty-one hospitals across Spain participated in the survey. These respondents represented sixteen Autonomous Communities (94%), with Andalusia, Catalonia, and the Valencian Community showing the highest response rates. La Rioja was the only region without representation. All respondents had experience in treating IFIs, with a mean of 22.86 ± 8.28 years of clinical practice. The survey achieved a 100% participation rate among invited specialists. The complete questionnaire and the responses provided by the respondents are shown in Table 1.

### 3.1. Pulmonary Aspergillosis (PA) in Critically Ill Patients

Responses provided by the respondents: (a) In patients with influenza/COVID-19 diagnosis is usually initiated when there is clinical deterioration that cannot be explained by any other cause upon admission to the ICU (67%). (b) In high-risk patients with clinical suspicion, bronchoscopic bronchoalveolar lavage (BAL) is usually performed to obtain lower respiratory tract samples, and galactomannan testing is requested to guide treatment (95%). (c) I start antifungal treatment without waiting for microbiological results when there is clinical deterioration in at-risk patients that cannot be explained by any other cause upon admission to the ICU (68%). (d) I am guided by the galactomannan result in tracheal aspirate to initiate treatment (27%).

Comments and discussion: Invasive pulmonary aspergillosis (IPA) is the most severe form of aspergillosis that typically occurs in patients with risk factors for developing aspergillosis, such as virus-induced ARDS, corticosteroid treatment, immunomodulatory therapies, and immunosuppressed states [10]. Furthermore, viral infections such as COVID-19 and influenza are widely recognized as predisposing factors for IPA [10]. According to Spanish practical recommendations for the diagnosis and management of PA associated with COVID-19 (CAPA) or influenza (IAPA), these diseases should be suspected as part of a differential diagnosis of respiratory superinfection in ICU patients with viral pneumonia who experience clinical deterioration not attributable to other factors [12]. Following these recommendations, diagnosis should be based on galactomannan determination in respiratory samples, direct microscopic examination, culture on selective media, and molecular detection of *Aspergillus* DNA by polymerase chain reaction (PCR), whenever available [12]. If possible, it is recommended to obtain samples from the lower respiratory tract using a flexible bronchoscope to collect a BAL specimen. Respiratory samples not obtained by fiberoptic bronchoscopy such as sputum, tracheal aspirate and non-bronchoscopic lavage (NBAL) are considered insufficient for diagnosis of this type of patient and are therefore not recommended. In addition, these recommendations suggest initiating antifungal treatment when clinical suspicion is high, and results are either delayed or inconclusive. Afterward, depending on the results, antifungal treatment can be modified or discontinued [12,13].

The results obtained from the survey support the consensus recommendations, suggesting that clinicians might know and guide the diagnosis and treatment of CAPA and IAPA according to them.

### 3.2. The Latest Epidemiological Studies on Aspergillus Resistance Conducted in Spain (ASPEIN I and ASPEIN II) Confirm the Presence of Azole-Resistant Aspergillus fumigatus

Answers provided by the respondents: (a) I request that the laboratory or another relevant institution assess azole susceptibility whenever *Aspergillus* spp. is identified in culture (70%). (b) I request that the laboratory or another relevant institution to determine resistance mutations when *Aspergillus* spp. is detected in culture (11%). (c) It is necessary to consider the possibility of azole resistance in patients who show no clinical improvement (87%). (d) Most observed azole resistance is believed to be of environmental origin (27%).

Comments and discussion: *Aspergillus fumigatus* is one of the main etiological agents of aspergillosis in Spain [14,15]. It belongs to a broader species complex that includes *Aspergillus fumigatus sensu stricto* and several cryptic species, which often differ in antifungal susceptibility [16]. Recently, the results of the ASPEIN surveillance study conducted in 29 Spanish hospitals were published [16]. This study aimed to assess the burden of azole resistance in 847 clinical *Aspergillus fumigatus* isolates obtained from 725 patients between 15 February and 14 May 2019. Most isolates originated from the lower respiratory tract (94.0%); 97.8% corresponded to *Aspergillus fumigatus sensu stricto* and 2.2% to cryptic species. Among the 847 isolates, 63 (7.4%) showed resistance to at least one azole, with resistance rates markedly higher in cryptic species than in *Aspergillus fumigatus sensu stricto* (94.7% vs. 5.5%). Moreover, amphotericin B resistance was observed exclusively in cryptic species. The authors concluded that antifungal resistance is present in Spain and that addressing it is essential, as resistance is associated with increased mortality.

Several guidelines have been developed to support the diagnosis and treatment of life-threatening diseases caused by *Aspergillus* spp., [17,18,19]. The 2018 ESCMID and GEMICOMED guidelines recommend performing antifungal susceptibility testing in patients with invasive aspergillosis (IA) in regions where antifungal resistance has been detected. In addition, these guidelines emphasize that antifungal resistance should be suspected when therapeutic failure occurs and when cryptic species are identified as causative agents of IA [17,19]. These recommendations are highly consistent with the results of the survey, suggesting that respondents are familiar with and adhere to published guidelines.

### 3.3. In Cases of Suspected Azole Resistance in a Patient Undergoing Treatment for Aspergillosis, What Strategy Would You Pursue?

Answers provided by the respondents: (a) Addition of another antifungal from a different class than the one currently being administered (40%). (b) Switching to a broad-spectrum antifungal class (44%). (c) Increasing the antifungal dose (0%). (d) Combined therapy with two new antifungals from different classes other than azoles (16%).

Comments and discussion: There is limited evidence available in the literature to guide optimal treatment decisions in cases of suspected azole resistance in patients with aspergillosis. However, the evidence has demonstrated that azole resistance is associated with increased mortality rates, particularly among patients admitted to the ICU [20,21,22]. A five-year retrospective cohort study carried out at 3 tertiary care medical centers in the Netherlands analyzed clinical characteristics and mortality rates of 196 patients with invasive aspergillosis (IA) [21]. The authors reported voriconazole resistance in 19% of the patients, increasing to 24% among those admitted to the ICU. Voriconazole resistance was associated with significantly higher mortality compared with susceptible cases, with mortality rates of 49% vs. 28% at day 42 (*p* = 0.017) and 62% vs. 37% at day 90 (*p* = 0.0038). Similar finding were reported in another retrospective cohort study conducted in a single ICU of a tertiary university hospital in the Netherlands [22]. A total of 136 patients were treated for suspected FI of which 28% had a positive culture for *Aspergillus fumigatus.* Azole resistance was identified in 26% of these patients admitted to the ICU while comparable rates (24%) correspond to all other departments in the hospital. Among patients with IA caused by azole-resistant *Aspergillus fumigatus*, 90-day mortality was 100% compared to 82% in azole-susceptible patients. The authors emphasized the importance of optimizing diagnostic strategies and reassessing empirical antifungal therapy in these settings [22].

According to the clinical guidelines published by the Study Group of FIs (GEMICOMED) of the Spanish Society of Infectious Diseases and Clinical Microbiology (SEIMC), treatment with amphotericin B (AIII) or combination therapy with voriconazole plus an echinocandin (CIII) is recommended for IA caused by cryptic species or voriconazole-resistant isolates (MIC > 2 mg/L) [17]. In addition, this guideline suggests that in areas with a rate of azole resistance > 10%, azole monotherapy should be avoided, proposing a change in the treatment strategy. Consistent with these recommendations, survey results suggest that clinicians adhere to current guidelines, with no clear preference between the two recommended regimens.

### 3.4. In the Treatment of Aspergillosis in Patients with Influenza

Answers provided by the respondents: (a) Isavuconazole and liposomal amphotericin B are recommended treatments (86%). (b) I would start it with a galactomannan + Bronchoalveolar Lavage (BAL) (81%). (c) Empirical treatment in the absence of laboratory tests remains valid when there is high clinical suspicion (76%). (d) I believe that prophylaxis must be done in ventilated critical patients (6%).

Comments and discussion: In cases of a positive *Aspergillus* culture in patients with severe viral pneumonia due to influenza, an individualized approach should be taken into account. First, it is essential to determine whether the patient is critically ill and has severe respiratory compromise, followed by prompt initiation of appropriate antifungal treatment [12]. If subsequent respiratory deterioration occurs, collecting new high-quality respiratory specimens, such as bronchoalveolar lavage, and initiating early treatment is recommended, since the influenza virus is a known risk factor for the development of invasive pulmonary aspergillosis (IPA) [12]. Mould-active azoles, such as voriconazole or isavuconazole, are the first-line antifungal agents for the treatment of influenza-associated pulmonary aspergillosis (IAPA), with liposomal amphotericin B as an alternative in settings where azole resistance is prevalent [23].

Additionally, data from the study published by Peral et al. indicate that in cases of severe pneumonia due to the influenza virus, bronchoscopy should be performed as soon as possible [12]. Similarly, a study published by Feys et al. demonstrated that, in the cases of IAPA in critically ill patients, BAL sampling for culture, galactomannan testing, and polymerase chain reaction (PCR) should be considered the cornerstone of diagnosis. Moreover, visual examination of the tracheobronchial tract during bronchoscopy is required to detect invasive *Aspergillus* (IA) tracheobronchitis. In addition, galactomannan testing in BAL fluid is considered the most valuable mycological tool to establish the diagnosis of IAPA [23]. Therefore, the results of both studies are consistent with the data obtained from this survey.

On the other hand, according to the study by Peral et al., there is no scientific evidence to support specific recommendations when a positive *Aspergillus* culture is obtained in patients with severe viral pneumonia due to Influenza [12]. However, data from our study indicate that more than three-quarters of experts were in favour of empirical treatment in the absence of laboratory confirmation when there is high clinical suspicion. This supports the hypothesis that the high morbidity and mortality associated with invasive pulmonary aspergillosis may be related to delayed initiation of treatment, emphasizing the importance of early therapy to improve outcomes. Regarding antifungal prophylaxis, it is not recommended in patients with severe influenza [12]. This finding is consistent with the data obtained in our survey, as only a very low percentage of experts supported prophylactic treatment in critically ill, mechanically ventilated patients.

### 3.5. Some Antifungals Do Not Reach Therapeutic Levels During the First Few Days of Administration. In This Situation, When Invasive Aspergillosis (IA) Is Suspected, Which Strategy Do You Consider Most Appropriate?

Answers provided by the respondents: (a) Add a broad-spectrum antifungal from a different class and wait for clinical improvement of the patient (5%). (b) Verify that the patient is not at risk of subtherapeutic antifungal levels due to drug interactions and maintain monotherapy (32%). (c) Add an antifungal from another class and perform therapeutic drug monitoring before returning to monotherapy (43%). (d) If therapeutic drug monitoring (TDM) for azoles is not available, I avoid using them (21%).

Comments and discussion: *Aspergillus* resistance to antifungal drugs should be suspected in any scenario of therapeutic failure (regardless of the isolated species) and when cryptic species are identified as causative agents of IA [17]. Every isolate obtained from an invasive infection should undergo antifungal resistance testing. Currently, antifungal susceptibility testing (AFST) remains the most reliable method for determining the most active antifungal agent and for detecting resistance in *Aspergillus* spp. However, the true global rates of antifungal resistance in these pathogens remain unknown, although they are reportedly low in Spain. Antifungal combination therapy is generally not recommended for the primary treatment of IA. However, in salvage therapy for refractory IA, adding a second agent to the initial regimen may be considered in some patients [17].

According to IDSA guidelines, an individualized approach that considers the speed, severity, and extent of the infection, the patient’s comorbidities, and excludes the emergence of a new pathogen is recommended [18]. The duration of antifungal therapy for invasive aspergillosis (IA) is not well defined. These guidelines generally recommend that treatment for invasive pulmonary aspergillosis (IPA) should continue for a minimum of 6–12 weeks, depending on disease severity [18].

Echinocandins are effective in salvage therapy (either alone or in combination) against IA. However, their routine use as monotherapy for the primary treatment of IA is not recommended. In the context of salvage therapy, an additional antifungal agent may be added to the current regimen, or antifungal agents from classes different from those used in the initial regimen may be combined [18]. In contrast, the clinical practice guidelines developed by García et al. highlight that the use of two or more antifungal drugs may also result in increased toxicity or drug–drug interactions [17].

For therapies in which drug interactions with azoles are anticipated, TDM is recommended to prevent therapeutic failure attributable to suboptimal drug exposure, and to minimize toxicities potentially associated with voriconazole [18].

TDM of antifungal agents is generally recommended, particularly in situations involving non-compliance, non-linear pharmacokinetics, inadequate absorption, a narrow therapeutic window, suspected drug interactions or unexpected toxicity [17]. Nevertheless, in Spain, a national survey distributed among colleagues of GEMICOMED found that TDM was not available in almost half of the hospitals, and samples were sent to reference hospitals [24].

IDSA guidelines also highlight the heterogeneity of the results found in published studies. Additional questions regarding optimal drug combinations, adequate dosing, pharmacokinetic interactions and potential toxicities associated with primary combination antifungal therapy require further investigation [18].

Overall, the findings of these studies and clinical guidelines are consistent with the results of this survey. Several hospitals in Spain do not have access to TDM, and there is no consensus among respondents regarding the most appropriate management strategy when antifungal agents fail to reach adequate serum levels during the first days of treatment.

### 3.6. In Your Opinion, Azole-Resistant Candida parapsilosis

Answers provided by the respondents: (a) Liposomal amphotericin B is one of the main treatment options (79%). (b) It is not associated with prior azole exposure (24%). (c) It is associated with higher mortality compared to non-resistant strains (43%). (d) I consider it an emerging concern in my clinical setting (59%).

Comments and discussion: *Candida parapsilosis* is one of the most common causative species of invasive candidiasis (IC) and candidemia, particularly among immunocompromised and critically ill patients [25]. In recent years, an increase in fluconazole-resistant strains has been reported, representing a growing global concern due to their high morbidity and mortality rates [25]. Consequently, *Candida parapsilosis* has been considered in the WHO fungal priority pathogen list [25,26].

In Spain, several fluconazole-resistant *Candida parapsilosis* outbreaks have been reported in regions such as Madrid and Castilla y Léon [27,28]. These reports emphasized the importance of implementing strict infection control measures to prevent further spread [27,28].

Reported mortality rates for IC infections caused by *Candida parapsilosis* ranged from 14.5% to 47.0%. However, several observational studies indicate that fluconazole-resistant *Candida parapsilosis* is not associated with higher mortality compared with fluconazole-susceptible strains [29,30]. These findings are consistent with the responses that were obtained in the survey.

Regarding the treatment of *Candida parapsilosis*, no guidelines or recommendations have been found in the literature. Nevertheless, the global guideline published in 2025 by the European Confederation for Medical Mycology (ECMM) in cooperation with the International Society of Human and Animal Mycology (ISHAM) and the American Society for Microbiology (ASM) provides several recommendations for the treatment of candidiasis. Echinocandins are recommended as a first-line therapeutic option [31]. In addition, L-AMB is strongly recommended in patients who cannot be treated with echinocandins due to proven or suspected drug resistance, treatment failure or intolerance. Moreover, this guideline also underscores the importance of considering local epidemiology when deciding treatment [31].

### 3.7. In Your Opinion, Candida auris

Answers provided by the respondents: (a) I recommend combination therapy (73%). (b) Echinocandins and liposomal amphotericin B are the main treatment options (68%). (c) It is associated with higher mortality compared to other forms of invasive candidiasis (IC) (76%). (d) I consider *Candida auris* an emerging threat in my clinical setting (38%).

Comments and discussion: *Candida auris* is an emerging multidrug-resistant pathogen that causes different nosocomial outbreaks in several countries around the world [32,33]. In Spain, several *Candida auris* outbreaks have been reported in several hospitals in Valencia [32,34,35,36,37]. However, in other geographic regions, such as Madrid, *Candida auris* has not been detected [28]. These epidemiological data are consistent with the fact that just 38% of the respondents stated that *Candida auris* is an emerging threat in their clinical setting.

Moreover, *Candida auris* exhibits high levels of antifungal resistance to fluconazole and voriconazole and variable susceptibility to amphotericin B and echinocandins [33,36]. In addition, multidrug resistance and even pan-resistance have been documented in some *Candida auris* isolates [33]. Thus, *Candida auris* is a life-threatening infection that is associated with high mortality rates (29–72%) due to its difficult antifungal management [26,33,36,38].

Treatment of *Candida auris* remains challenging. However, echinocandins are currently recommended as a first-line therapeutic option [31]. If the patient is colonized or has had a history of infection with echinocandin-resistant strains, liposomal amphotericin B is moderately recommended [31]. Nevertheless, during some Spanish outbreaks, L-AmB has been added to echinocandin treatment in a high percentage of patients (35.4%) when persistent candidemia was observed [36]. In our survey, 73% of the respondents recommended combination therapy, suggesting that it may be more widely used in the Spanish setting.

### 3.8. In Which Situations Would You Initiate Antifungal Treatment for Invasive Candidiasis (IC)?

Answers provided by the respondents: (a) I do not initiate treatment until culture results are available and positive (2%). (b) I rely on B-D-glucan testing to guide my decision (35%). (c) I initiate treatment when the Candida score is greater than 3 (73%). (d) I start treatment in patients with persistent fever, poor clinical evolution, and no response to antibiotics, even before microbiological results are available (78%).

Comments and discussion: IC is one of the most challenging and potentially fatal infections in critical care [39,40]. Recently, there has been a worldwide epidemiological trend towards decreasing proportions of *Candida albicans.* However, the proportion of non-*albicans Candida* spp. as the main causative agent has increased [39,40]. The distribution of non-*albicans Candida* spp. varies widely across geographic regions [39]. In Spain, the CANDIMAD multicentre surveillance study carried out between 2020 and 2022 analysed the epidemiology and antifungal resistance of 766 *Candida* isolates from 686 patients [28]. During the described period of time, *C. albicans* remained the most frequent species followed by *C. parapsilosis* complex fluconazole-susceptible and *C. glabrata.* Nevertheless, there was a significant increase in fluconazole-resistant *C. parapsilosis* [28].

Timely and accurate diagnosis could enhance treatment success for critically ill patients with IC [40]. Accordingly, the European Confederation for Medical Mycology (ECMM) published in 2025 some clinical recommendations for the management of IC [31]. This guideline moderately recommends initiating empirical antifungal therapy in patients presenting with septic shock or patients with clinical deterioration who exhibit additional risk factors for candidemia, including prolonged ICU stay, presence of an indwelling vascular catheter or documented colonization by *Candida* species [31]. Furthermore, serum β-D-glucan (BDG) testing for diagnosing IC and candidemia is moderately recommended. However, they highlighted that the diagnosis should not be exclusively based on BDG testing [31].

In addition, the Spanish guideline developed in 2011 by the SEIMC also provides recommendations for the treatment of IC in critically ill patients [41]. Prophylactic treatment of IC in critically ill patients is recommended in high-risk patients identified based on the degree of colonization or on the presence of specific risk factors, such as the Candida score [41,42,43]. Additionally, this guideline underscored the importance of the timely initiation of antifungal therapy, as delayed treatment is associated with increased mortality rates [41].

Overall, our survey findings suggest that clinicians generally comply with established clinical guidelines. These results highlight the critical need to strengthen early and accurate diagnostic strategies to enable targeted antifungal therapy, minimize unnecessary antifungal exposure, and reduce the risk of antifungal resistance.

### 3.9. What Is Your Opinion Regarding Antifungals Drug Monitoring in Critically Ill Patients?

Answers provided by the respondents: (a) In practice, TDM is difficult to implement adequately, or results are not obtained in a timely manner (79%). (b) High-dose corticosteroids can reduce azole concentrations (37%). (c) Monitoring isavuconazole may be advisable in specific situations, such as in obese patients with a BMI > 25 kg/m^2^ or when extracorporeal membrane oxygenation (ECMO) or renal replacement therapies (RRT) are used (84%). (d) The use of ECMO and RRT may be associated with low azole levels (90%).

Comments and discussion: Therapeutic drug monitoring (TDM) of antifungals has been postulated as a useful tool to determine antifungal dosage, monitor therapeutic efficacy and minimize drug-related toxicity [24]. According to clinical guidelines, TDM is generally recommended, especially in critically ill patients or patients at risk of low or high drug exposures, non-linear pharmacokinetics, narrow therapeutic index, suspected drug–drug interactions, or unexpected toxicity [17,31]. Commonly, TDM should be considered when using itraconazole, posaconazole or voriconazole [44]. However, TDM is also recommended in patients with variable pharmacokinetics, including critically ill patients, treated with echinocandins or isavuconazole [44,45]. Moreover, recent real-world studies indicate that conditions such as obesity, renal replacement therapy and extracorporeal membrane oxygenation are linked to suboptimal isavuconazole levels [45,46,47].

In Spain, a national survey distributed among colleagues of GEMICOMED reported that TDM was not available in almost half of the hospitals and samples were sent to reference hospitals [24]. However, respondents stated that it was routinely carried out for antifungals such as voriconazole [24]. This limited on-site laboratory availability, in addition to delayed turnaround times, highlights some of the barriers to the implementation of TDM, which may reduce its clinical usefulness in real-time drug monitoring.

Our survey results align closely with published guidelines and recommendations, reinforcing their applicability in the real-world setting in Spain.

### 3.10. In Your Opinion, Regarding Intra-Abdominal Candidiasis (IAC)

Answers provided by the respondents: (a) Echinocandins are associated with the development of resistance in *Candida glabrata*, especially in this location (57%). (b) Pathophysiological changes in critically ill patients especially affect water-soluble drugs, reducing their penetration into the peritoneal fluid (78%). (c) *Candida glabrata* is increasing in incidence in this condition (73%). (d) This infection is frequently underdiagnosed (79%).

Comments and discussion: Intra-abdominal candidiasis (IAC) is caused by an overgrowth of *Candida* spp. within the abdominal cavity [48]. It accounts for 10–30% of all intra-abdominal infections diagnosed in the ICU and is associated with significantly higher morbidity and mortality rates [49]. Moreover, IAC may be associated with, or occur in the absence of candidemia, defined as the presence of *Candida* spp. in the blood [48]. Recently, there has been an epidemiological shift from *Candida albicans* to non-albicans species, such as *Candida glabrata* or *Candida auris*, which often present antifungal drug resistance, representing a growing threat to human health [48,50]. Consequently, some of these species have been included in the WHO fungal priority pathogen list [26]. Moreover, it has been revealed by polymerase chain reaction (PCR) testing that *C. glabrata* infections are often underdiagnosed [48].

A prospective study carried out between 2019 and 2021 in patients admitted to 16 hospitals in the Madrid area monitored the epidemiology and antifungal susceptibility of *Candida* spp. from blood cultures and intra-abdominal samples following the EUCAST E.Def 7.3.2 procedure [51]. From 2107 *Candida* isolates collected from blood and intra-abdominal samples, five species (*C. albicans*, *C. glabrata*, *C. parapsilosis*, *C. tropicalis*, and *C. krusei*) accounted for 96.9% of cases, and *C. auris* was not detected. Overall, resistance rates were 8.6% for fluconazole and 0.7% for echinocandins. Nevertheless, intra-abdominal samples of *Candida glabrata* showed higher resistance rates. Cross-resistance to echinocandins and azoles in *Candida glabrata* is becoming a concern [51].

Moreover, it is important to consider that this infection occurs in the peritoneal cavity. Penetration of antifungals into this cavity is particularly essential, especially in critically ill patients, in whom pathophysiological alterations may compromise drug distribution [52]. Several studies have demonstrated that echinocandin exposure is suboptimal in critically ill patients and thus, dose adjustments would be necessary to adequately treat IAC [44,53,54]. Consequently, the use of therapeutic drug monitoring (TDM) is recommended to optimize drug concentrations and effectiveness in critically ill patients and in patients at risk of suboptimal antifungal exposure [44,53].

Our survey results corroborate these findings, revealing comparable challenges and priorities among clinicians.

### 3.11. I Initiate Antifungal Treatment in Intra-Abdominal Candidiasis (IAC)

Answers provided by the respondents: (a) I would like to request the determination of B-D-glucan in peritoneal fluid (60%). (b) I rely on the isolation of yeasts in peritoneal fluid to guide treatment (57%). (c) I initiate therapy only when the Candida score is positive (6%). (d) In the immediate postoperative period of suture dehiscence in a situation of septic shock (92%).

Comments and discussion: The diagnosis and management of invasive candidiasis (IC) have greatly improved in recent years. However, its diagnosis and treatment remain a challenge for critical care specialists, and it is often accompanied by bacterial cross-infections [55]. To optimize the diagnosis and treatment of the Working Group on Perioperative Infections of IAC, the Spanish Society of Anaesthesiology, Resuscitation and Pain Management (GTIPO-SEDAR) has published several recommendations based on multidisciplinary expert opinion [55]. Upon suspicion of IAC, the experts recommended the determination of B-D-glucan in plasma and peritoneal fluid. Moreover, the experts also recommend analyzing abdominal fluid using molecular tests such as Polymerase Chain Reaction (PCR) to identify species, such as *C. glabrata,* which are associated with higher antifungal resistance [55]. In the survey, 60% of the respondents stated that they would like to have the determination of B-D-glucan in peritoneal fluid which is aligned with the aforementioned recommendations. In addition, just 6% of the respondents commented that they initiate therapy only when the Candida score is positive, which is consistent with evidence showing that B-D-glucan determination outperforms the Candida score for early diagnosis [56]. Finally, a narrative review highlights the importance of early antifungal treatment in critically ill patients undergoing abdominal surgery, particularly those with septic shock or postoperative complications, which aligns with the current findings [48].

### 3.12. Regarding Mucor Infection in Critically Ill Patients with Severe Viral Lung Infection

Answers provided by the respondents: (a) I would like to request the determination of C-reactive protein (CRP) in bronchoscopic bronchioalveolar lavage (BAL) (76%). (b) The treatment of choice is liposomal amphotericin B (87%). (c) Diagnosis is primarily based on culture from bronchoscopic BAL samples (46%). (d) I consider this infection rare in our setting, but it should remain on our radar (94%).

Comments and discussion: Mucormycosis is a rare life-threatening FI that tends to progress rapidly and is associated with high morbidity and mortality. Early diagnosis and urgent multidisciplinary intervention are extremely important to maximize survival rates. To facilitate clinical decision-making, the European Confederation of Medical Mycology (ECMM), together with the Mycoses Study Group Education & Research Consortium (MSG ERC), has developed a guidance document [57].

Diagnosis of mucormycosis should be rapid, combining imaging, histopathology and culture of deep tissue samples from the affected site, such as BAL fluid in cases of pulmonary involvement.

Once diagnosed, early complete surgical treatment in addition to systemic antifungal treatment is strongly recommended. First-line drug treatment with high-dose liposomal amphotericin B is strongly recommended across all patterns of organ involvement. Moreover, isavuconazole is also recommended for first-line treatment with moderate strength. Strongly recommended treatment of mucormycosis in critically ill patients admitted to the ICU includes correction of the underlying conditions where feasible, source control, appropriate antifungal therapy, and relevant supportive care [57].

Survey responses demonstrated that clinicians usually follow this guideline regarding drug treatment and confirm that mucormycosis remains a rare infection in Spain.

### 3.13. In My Hospital, I Have Access to

Answers provided by the respondents: (a) Galactomannan testing and bronchoalveolar lavage (BAL) (90%). (b) Real-time antifungal Therapeutic Drug Monitoring (TDM) (16%). (c) Lateral Flow assays (44%). (d) 24/7 fibreoptic bronchoscopy (87%).

Comments and discussion: Early diagnosis and more precise species identification of IFI are of crucial importance to initiate prompt antifungal therapy, as delayed diagnosis is associated with significantly higher mortality, especially in immunocompromised patients, as well as in critically ill individuals admitted to ICUs [3,58]. Nevertheless, accurate IFI diagnosis remains a challenge.

Currently, conventional techniques such as fungal culture, direct microscopy, and histopathology remain the standard of care for diagnosing IFI [3]. Despite enabling pathogen identification and antifungal susceptibility testing (AFST), these techniques provide suboptimal sensitivity and delayed reporting. However, novel techniques such as serological assays (β-D-glucan and galactomannan detection), polymerase chain reaction (PCR), MALDI-TOF, and lateral-flow assays have been developed to overcome these limitations [3].

In Spain, according to the obtained responses, access to rapid diagnostics and TDM remains heterogeneous. However, the presence of galactomannan testing and BAL, in addition to 24/7 fibreoptic bronchoscopy in most of the hospitals surveyed, supports guidelines-recommended diagnostic practices.

In contrast, although TDM of antifungal agents is generally recommended (***AII***) by the clinical guidelines published by GEMICOMED, its implementation in the Spanish setting remains heterogeneous. This finding contrasts with the results obtained from a survey completed by 22 clinicians in Spain, where 45.5% of the respondents stated that TDM was not available in their center and, therefore, samples were sent to reference hospitals, while 9.0% reported that it was neither available onsite nor sent to a central laboratory [24].

Overall, these findings underscore the importance of ensuring access to advanced diagnostic and monitoring technologies in Spanish hospitals to enable timely detection and treatment of IFI. The presence of such tools is essential to improve clinical outcomes and align practice with current guideline-recommended standards.

## 4. Discussion

This study describes the current situation and strategies for diagnosing and managing IFI in critically ill patients in Spain and has the strength of gathering expert opinions from intensivists through a survey, thereby reflecting real-world clinical practice. The observed variability in survey responses is considered justifiable due to the lack of consistent randomized clinical trials providing robust evidence. Most of the existing evidence in the field of fungal infections comes from studies conducted in immunocompromised patients, largely within hematology, and these results have often been extrapolated to critically ill patients, who, although sharing some similarities, present a distinct clinical profile, as evidenced by our detailed analysis of each survey question. Another notable strength is the high representativeness of most regions of Spain, enhancing the generalizability of the findings. Although inviting participants to the survey may introduce a potential selection bias, it should be emphasized that the respondents were intensivists specialized in infectious diseases and recognized as key opinion leaders within their respective units. Nevertheless, the reliance on self-reported data and the expert-survey design may limit external validity, and the findings should be interpreted as reflecting informed expert perspectives rather than prospective clinical outcome data.

Based on these results, it can be concluded that most respondents agree on the following points: (1) In case of PA in high-risk patients, when clinically suspected, BAL is usually performed to obtain samples from the lower respiratory tract, requesting galactomannan to guide treatment. (2) It is necessary to begin considering the presence of azole resistance in patients who show no clinical improvement, and its management should be based on an alternative broad-spectrum antifungal class. (3) The use of isavuconazole and liposomal amphotericin B is recommended for the treatment of aspergillosis in patients with influenza. (4) It would be advisable to monitor isavuconazole in specific situations. Furthermore, the use of ECMO and RRT may be associated with low levels of azoles. (5) Antifungal treatment for IAC must be initiated immediately after surgery in cases of suture dehiscence in septic shock. (6) Mucor infection in critically ill patients with severe viral lung infection is rare, but the treatment of choice is liposomal amphotericin B.

## Figures and Tables

**Table 1:** Questions included in the survey and the responses provided by the respondents.

Questions	Answers N (%)
1. Pulmonary aspergillosis (PA) in critically ill patients *:	
In patients with influenza/COVID-19 diagnosis is usually initiated when there is clinical deterioration that cannot be explained by any other cause upon admission to the ICU.	42 (67%)
In high-risk patients with clinical suspicion, bronchoscopic bronchoalveolar lavage (BAL) is usually performed to obtain lower respiratory tract samples, and galactomannan testing is requested to guide treatment.	60 (95%)
I start antifungal treatment without waiting for microbiological results when there is clinical deterioration in at-risk patients that cannot be explained by any other cause upon admission to the ICU.	43 (68%)
I am guided by the galactomannan result in tracheal aspirate to initiate treatment.	17 (27%)
2. The latest epidemiological studies on *Aspergillus* resistance conducted in Spain (ASPEIN I and ASPEIN II) confirm the presence of azole-resistant *Aspergillus fumigatus* *.	
I request that the laboratory or another relevant institution assess azole susceptibility whenever *Aspergillus* spp. is identified in culture.	44 (70%)
I request the laboratory or another relevant institution to determine resistance mutations when *Aspergillus* spp. is identified in culture.	7 (11%)
It is necessary to consider the possibility of azole resistance in patients who show no clinical improvement.	55 (87%)
Most observed azole resistance is believed to be of environmental origin.	17 (27%)
3. In cases of suspected azole resistance in a patient undergoing treatment for aspergillosis, what strategy would you pursue?	
Addition of another antifungal from a different class than the one currently being administered.	25 (40%)
Switching to a broad spectrum antifungal class.	28 (44%)
Increasing the antifungal dose	0 (0%)
Combined therapy with two new antifungals from different classes other than azoles.	10 (16%)
4. In the treatment of aspergillosis in patients with influenza *:	
Isavuconazole and liposomal amphotericin B are recommended treatments	54 (86%)
I would start it with a galactomannan + Bronchoalveolar Lavage (BAL)	51 (81%)
Empirical treatment in the absence of laboratory tests remains valid when there is high clinical suspicion	48 (76%)
I believe that prophylaxis must be done in ventilated critical patients	4 (6%)
5. Some antifungals do not reach therapeutic levels during the first few days of administration. In this situation, when invasive aspergillosis (IA) is suspected, which strategy do you consider most appropriate?	
Add a broad-spectrum antifungal from a different class and wait for clinical improvement of the patient	3 (5%)
Verify that the patient is not at risk of subtherapeutic antifungal levels due to drug interactions and maintain monotherapy	20 (32%)
Add an antifungal from another class and perform therapeutic drug monitoring before returning to monotherapy	27 (43%)
If therapeutic drug monitoring (TDM) for azoles is not available, I avoid using them.	13 (21%)
6. In your opinion, azole-resistant *Candida parapsilosis* *:	
Liposomal amphotericin B is one of the main treatment options	50 (79%)
It is not associated with prior azole exposures	15 (24%)
It is associated with higher mortality compared to non-resistant strains	27 (43%)
I consider it an emerging concern in my clinical setting	37 (59%)
7. In your opinion, *Candida auris* *:	
I recommend combination therapy	46 (73%)
Echinocandins and liposomal amphotericin B are the main treatment options	43 (68%)
It is associated with higher mortality compared to other forms of invasive candidiasis (IC)	48 (76%)
I consider *Candida auris* an emerging threat in my clinical setting.	24 (38%)
8. In which situations would you initiate antifungal treatment for invasive candidiasis (IC)? *	
I do not initiate treatment until culture results are available and positive	1 (2%)
I rely on B-D-glucan testing to guide my decision	22 (35%)
I initiate treatment when the Candida score is greater than 3	46 (73%)
I start treatment in patients with persistent fever, poor clinical evolution, and no response to antibiotics, even before microbiological results are available	49 (78%)
9. What is your opinion regarding antifungal drug monitoring in critically ill patients? *	
In practice, TDM is difficult to implement adequately, or results are not obtained in a timely manner.	50 (79%)
High-dose corticosteroids can reduce azole concentrations	23 (37%)
Monitoring isavuconazole may be advisable in specific situations, such as in obese patients with a BMI > 25 m^2^ or when extracorporeal membrane oxygenation (ECMO) or renal replacement therapies (RRT) are used	53 (84%)
The use of ECMO and RRT may be associated with low azole levels	57 (90%)
10. In your opinion, regarding intra-abdominal candidiasis (IAC) *:	
Echinocandins are associated with the development of resistance in *Candida glabrata*, especially in this location.	36 (57%)
Pathophysiological changes in critically ill patients especially affect water-soluble drugs, reducing their penetration into the peritoneal fluid	49 (78%)
*Candida glabrata* is increasing in incidence in this condition	46 (73%)
This infection is frequently underdiagnosed	50 (79%)
11. I initiate antifungal treatment in intra-abdominal candidiasis (IAC) *:	
I would like to request the determination of B-D-glucan in peritoneal fluid	38 (60%)
I rely on the isolation of yeasts in peritoneal fluid to guide treatment	36 (57%)
I initiate therapy only when the Candida score is positive	4 (6%)
In the immediate postoperative period of suture dehiscence in a situation of septic shock	58 (92%)
12. Regarding Mucor infection in critically ill patients with severe viral lung infection *:	
I would like to request the determination of C-reactive protein (CRP) in bronchoscopic bronchioalveolar lavage (BAL)	48 (76%)
The treatment of choice is liposomal amphotericin B	55 (87%)
Diagnosis is primarily based on culture from bronchoscopic BAL samples	29 (46%)
I consider this infection rare in our setting, but it should remain on our radar	59 (94%)
13. In my hospital, I have access to *:	
Galactomannan testing and bronchoalveolar lavage (BAL)	57 (90%)
Real-time antifungal therapeutic drug monitoring (TDM)	10 (16%)
Lateral Flow assays	28 (44%)
24/7 fiberoptic bronchoscopy	55 (87%)

* Multiple answers were permitted.

## Data Availability

Data supporting reported results may be presented if necessary. All data generated or analysed during this study are included in this published article.
